# Genetic association of wool quality characteristics in United States Rambouillet sheep

**DOI:** 10.3389/fgene.2022.1081175

**Published:** 2023-01-23

**Authors:** Gabrielle M. Becker, Julia L. Woods, Christopher S. Schauer, Whit C. Stewart, Brenda M. Murdoch

**Affiliations:** ^1^ Department of Animal, Veterinary and Food Science, University of Idaho, Moscow, ID, United States; ^2^ Hettinger Research Extension Center, North Dakota State University, Hettinger, ND, United States; ^3^ Department of Animal Science, University of Wyoming, Laramie, WY, United States

**Keywords:** 60S ribosomal protein L17-like, ABCC8, central performance ram test, GWAS, sheep production

## Abstract

**Introduction:** Fine wool production is an important source of revenue, accounting for up to 13% of total revenue in extensively managed wool sheep production systems of the United States. The Rambouillet are a predominant breed that excels in wool quality characteristics. Understanding the genetic basis of wool quality characteristics would aid in the development of genomic breeding strategies to facilitate genetic improvement.

**Methods:** Wool characteristics and DNA were collected for rams enrolled in the North Dakota State University and University of Wyoming annual central performance ram tests over a three-year period (2019–2021, *N* = 313). The relationships of wool quality characteristics including grease fleece weight adjusted 365 days (wt. 365 adj.), clean fleece wt. 365 adj., staple length 365 adj., average fiber diameter, face wool cover, amount of skin wrinkles and belly wool were evaluated through genome-wide association studies (GWAS), Pearson correlation and ANOVA.

**Results:** The GWAS identified four genome-wide significant genetic markers (*p*-value <1.19e-06) and five chromosome-wide significant markers (*p-*value <1.13e-05) on chromosomes 1, 2, 4, 15, and 19. Significant markers were associated with genes notable for relevant wool biological functions, including the gene *ABCC8* which codes for SUR1, an ATP-sensitive potassium channel known to affect hair growth and 60S ribosomal protein L17-like, previously found to be expressed during follicle formation. The strongest Pearson correlation coefficients were identified between clean fleece wt. 365 adj. and grease fleece wt. 365 adj. (*r* = 0.83) and between clean fleece wt. 365 adj. and staple length 365 adj. (*r* = 0.53). Additionally, clean fleece wt. 365 adj. was correlated with final body weight (*r* = 0.35) and scrotal circumference (*r* = 0.16). Staple length 365 adj. (*p*-value = 5e-04), average fiber diameter (*p*-value = .0053) and clean fleece wt. 365 adj. (*p*-value = .014) were significantly associated with belly wool score.

**Discussion:** The results of this study provide important insight into the relationships between wool quality characteristics and report specific markers that Rambouillet sheep producers may use to help inform selection and breeding decisions for improved wool quality.

## 1 Introduction

Rambouillet are a predominant United States breed in extensive and semi-extensive production systems. This breed is commonly used in arid and semi-arid rangeland systems as a dual-purpose breed excelling in both fine wool and meat products (Lupton et al., 2007; Burton et al., 2015). Wool production is an important source of strategically timed revenue for sheep producers and fine wool receipts account for up to 13% of total revenue from sheep production in the United States (Liver Marketing Information Center, 2016; Murphy et al., 2019). Wool quality characteristics are well studied in Rambouillet and other fine-wool breeds, yet the genomic mechanisms underpinning these traits are still poorly defined and underutilized in genomic breeding strategies.

Wool quality is driven by clean fleece weight and fiber diameter (Khan et al., 2012) but many characteristics can contribute to the overall economic worth. Central performance ram tests have been developed as a way to systematically evaluate ram growth and performance traits under comparable environmental conditions with demonstration and outreach value for sheep producers (Shelton et al., 1954; Burton et al., 2015). Ram tests are held annually at North Dakota State University (NDSU) and the University of Wyoming (UWY) to evaluate Rambouillet and other wool breeds enrolled by local sheep producers.

Much progress has been made in sheep production through the identification and utilization of genetic markers for disease susceptibility risk or carrier identification (Westaway et al., 1994; Cockett et al., 1999), reproduction traits (Ivanova et al., 2021) and carcass and milk traits (Clop et al., 2006; Selvaggi et al., 2015). Wool quality characteristics have been previously estimated to be moderately to highly heritable, indicating that trait variation is greatly influenced through genetic effects and progress may be made through genomic selectin (Bromley et al., 2000; Burton et al., 2015). Despite such promising heritability estimates, few validated markers exist for use with Rambouillet genomic breeding strategies.

The aim of this study was to utilize data collected during NDSU and UWY central performance ram tests over a three-year period to characterize relationships between traits and with genomic single nucleotide polymorphism (SNP) markers. Pearson correlation and analysis of variance (ANOVA) testing were conducted with wool characteristics grease fleece weight adjusted to 365 days (wt. 365 adj.), clean fleece wt. 365 adj., staple length 365 adj., average fiber diameter, face wool score, skin wrinkle and belly wool scores and production traits including initial and final weights, 140-day average daily gain (ADG) and scrotal circumference. Wool traits were evaluated in individual GWAS with 50 k genotype data to identify markers for use in genomic breeding strategies.

## 2 Materials and methods

### 2.1 Ram test protocols

Ram lambs 7 ± 3 months of age from regional (WY, ND, SD, MT, CO) seedstock producers were brought to the University of Wyoming—Laramie Research and Extension Center (Laramie, WY; 41°17′ N, −105°40′ W) or North Dakota State University—Hettinger Research and Extension Center (Hettinger, ND; 46°01′ N, −102°65’ W). Initial body weights were measured and animals were managed as one cohort. Rams were provided *ad libitum* textured diets (15%–17% crude protein, dry matter basis; 68%–73% total digestible nutrients dry matter basis) for 140 days in a dry-lot management system.

Rams were shorn after a 7–10-day acclimation period and once again at the conclusion of the 140-day feeding period. Upon conclusion of the performance test, scrotal circumference was obtained and wool staple length was measured on shoulder, side and britch, the three measurements averaged, and adjusted from 140-day to 365-day lengths in accordance with the standard practice of the National Sheep Improvement Program (NSIP) for this trait (Wilson and Morrical, 1991). This was calculated by dividing the average staple length by 140, to calculate staple length/day, and then multiplying by 365.

The presence of belly-type wool (belly wool) was scored from 1 to 4. In brief, belly wool is that which grows on the ventral region of the sheep and is characterized as uneven, tender in tensile strength, and compressed in staple length. Phenotypic selection pressure against this “belly wool” fiber type extending beyond the ventral portions of the sheep has been employed to avoid the resultant reduction in overall wool quality (Lupton et al., 2007; Naidoo et al., 2016). Thus, a subjective 1 to 4 scoring system was assigned in the fleece to rams at the end of the test period where: 1 = belly wool restricted to ventral portion, 2 = belly wool restricted to lower 1/3rd of side of fleece, 3 = belly wool extending from 1/3rd to ½ of the side of fleece, 4 = wool extending above ½ of the side of the fleece. Rams were scored linearly between these thresholds with a score of 1 being the minimum and a score of 4 being the highest possible (e.g., a ram with belly wool extending midway between the ventral portion and 1/3 of the side would be scored 1.5).

Similarly, a subjective 1 to 4 scoring system was assigned for face cover where: 1 = no wool cover over top of the head and on the side of muzzle, nor between eyes and ears, 2 = minimal wool cover over top of the head and on side of muzzle, and between eyes and ears, 3 = moderate wool cover over top of the head and on side of muzzle, and between eyes and ears, 4 = heavy wool cover over top of the head and on side of muzzle, and between eyes and ears. A skin wrinkle score was assessed once wool was shorn, where: 1 = no observable wrinkles on body surface 2 = minimal observable wrinkles on body surface 3 = moderate observable wrinkles on body and 4 = heavy wrinkles on body surface.

At shearing, whole fleeces were weighed and then individually cored in a custom-built apparatus (Gerbers of Montana, Inc., Great Falls, MT) consisting of 16 coring tubes (2.2 cm in diameter) that were plunged into and retracted from compacted fleeces by hydraulic cylinders. Cores were split into duplicate 25-g sub-samples for each animal to determine average laboratory scoured yield (American Society for Testing and Materials, 1990) from which clean fleece weight (CFW) was also estimated (Grease Fleece Weight x LSY). Grease and clean fleece weights were adjusted from 140-day to 365-day lengths in the same manner described for staple length (Wilson and Morrical, 1991). A single washed core subsample was analyzed on an Optical-based Fibre Diameter Analyser 2000 (OFDA; BSC Electronics Pty. Ltd., Attadale, Western Australia) to quantify average fiber diameter (A-FD) (IWTO, 2013).

### 2.2 Statistical evaluation of wool characteristics

Wool characteristics analyzed included grease fleece weight adjusted to 365 days (wt. 365 adj.) and clean fleece wt. 365 adj. (pounds), average fiber diameter (micron), staple length 365 adj. (inches), face wool score and skin wrinkle score as continuous variables and belly wool score as a categorical variable. All traits were tested for normality using the Shapiro Wilks test in R version 4.2.1 (R Core Team 2021); face and skin wrinkle scores were transformed using a log10 transformation to improve normality. Belly wool scores were grouped into the variable “belly wool category” with rams with no belly-type wool comprising category one (*n* = 273), rams with belly wool on less than one-third of the side comprising category two (*n* = 25) and rams with belly wool from one-third of the side to over one-half of the side comprising category three (*n* = 15). Wool characteristics were analyzed against other production traits including initial body weight and final body weight (pounds), 140-day average daily gain (140 days ADG) and scrotal circumference (centimeters).

The relationships between continuous wool quality characteristics and production traits were investigated with Pearson correlation to describe the strength and direction of linear correlation. One-way analysis of variance (ANOVA) testing was utilized to compare production trait and continuous wool quality trait means between belly wool categories. All ANOVA tests were further analyzed with *post hoc* Tukey HSD testing to compare *p*-values between categories (Abdi and Williams, 2010). Ram test location (North Dakota or Wyoming) was evaluated by Welch’s two-sample *t*-test and ram test year (2019, 2020, 2021) was tested by ANOVA to determine significance for potential GWAS fixed effects (Table 1). Pearson correlation testing were conducted and visualized using the corrplot package in R (Wei and Simko, 2021). ANOVA and Tukey HSD were conducted with the rstatix package and visualized with ggplot2, ggpubr and patchwork in R (Kassambara 2020a; Kassambara 2020b; Pedersen 2020).

**TABLE 1:** *p*-values for location and year against wool characteristics. Wool characteristics were tested against location (NDSU or UWY) with *t*-test and year (2019, 2020, 2021) with ANOVA.

	Location (*t*-test)	Year (ANOVA)
Grease Fleece Wt. 365 adj.	7.23E-04*	7.21E-01
Clean Fleece Wt. 365 adj.	2.39E-03*	1.81E-03*
Staple Length 365 adj.	1.79E-10*	1.29E-01
Average Fiber Diameter	3.55E-01	4.93E-03*
Face Wool Score	1.23E-05*	1.27E-01
Skin Wrinkle Score	<2.2e-16*	4.29E-02*

*indicates significant *p*-values of covariates included as fixed effects in EMMAX GWAS for trait model.

### 2.3 DNA genotyping and quality control

Ram DNA samples were extracted from either whole blood samples or ear tissue samples stored in tissue sampling units (TSU) collected by University of Wyoming or North Dakota State University personnel. DNA was isolated from blood at the University of Idaho using the phenol-chloroform method described previously (Sambrook et al., 1989) and TSU were provided to AgResearch for DNA extraction. Ram DNA samples were genotyped with either the Applied Biosystems™ Axiom™ Ovine Genotyping Array (50K) consisting of 51,572 single nucleotide polymorphism (SNP) markers (Thermo Fisher Scientific, catalog number 550898) or the AgResearch Sheep Genomics 60K SNP chip consisting of 68,848 SNP markers (GenomNZ, AgResearch, New Zealand). Duplicate markers designed for the same genomic position within a panel were filtered to retain the marker with the highest call rate (CR). Compatible markers were matched by marker name and genome position resulting in a consensus dataset of 44,431 markers in common between the genotype platforms (Davenport et al., 2020). Plink v1.9 was used to merge genotype array data and correct markers designed for opposite strands (Purcell et al., 2007; https://pngu.mgh.harvard.edu/purcell/plink/). Markers were filtered for quality control in the following order: non-autosomal markers (1,019 SNPs), markers with a call rate (CR) <90% (87 SNPs), markers with a minor allele frequency (MAF) <0.01 (1,407 SNPs) and markers with Hardy-Weinberg Equilibrium *p*-values <1e-50 (30 SNPs) were excluded, for a total of 41,888 high-quality autosomal SNPs retained for final analyses. All rams had a CR of 95% or greater.

### 2.4 Principal component analysis

Principal component analysis (PCA) was carried out to investigate population structure. Analysis was conducted with plinkv1.9 and visualized with the package ggplot2 in R (Purcell et al., 2007; https://pngu.mgh.harvard.edu/purcell/plink/; Kassambara 2020b; R Core Team 2021). Principal components were plotted PC1 (x-axis) *versus* PC2 (y-axis). Separate plots were generated for each continuous wool characteristic and rams were color-coded on a gradient scale to indicate their position within the trait distribution.

### 2.5 Genome-wide association studies

Continuous wool characteristics were evaluated in genome-wide association studies (GWAS) using the Efficient Mixed-Model Association eXpedited (EMMAX) in SNP and Variation Suite™ v8.9.1 (Golden Helix, Inc., Bozeman, MT, www.goldenhelix.com). The EMMAX models estimated the proportion of variance explained (PVE) for each marker as previously described (Kang et al., 2010). Each trait was initially tested in additive, dominant and recessive inheritance models to identify the model of best fit to be carried through for final analysis. A genomic relationship matrix was fitted as a random effect to account for population structure and sample relatedness in each model (Kang et al., 2010). Ram test location and ram test year were fitted as fixed effects as warranted by *t*-test or ANOVA *p*-value for each trait (Table 1) and GWAS results were visualized with the CMplot package in R (Yin 2022). Genome-wide significance was determined by the Bonferroni threshold (*p*-values <1.19e-06) and a chromosome-wide significance threshold was determined by Bonferroni-adjustment of the number of markers on the largest chromosome (4,412 markers; *p*-values <1.13e-05). The trait distributions of significant markers were visualized using boxplot figures and significance was further evaluated through analysis of covariance (ANCOVA) and Tukey HSD testing in R with the rstatix, ggplot2, ggpubr, multcomp and patchwork packages (Hothorn et al., 2008; Abdi and Williams, 2010; Kassambara 2020a; Kassambara 2020b; Pedersen 2020; R Core Team 2021). Each ANCOVA model included the same covariate(s) as included in the corresponding GWAS model.

### 2.6 Genomic context of significant markers

The genomic contexts of significant markers were investigated using GenomeBrowser in NCBI (NCBI Resource Coordinators 2016) for the reference genome ARS-UI_Ramb_v2.0 (Davenport et al., 2022). For each genome-wide and chromosome-wide significant SNP, the reference sequence comprising 100 kb upstream and 100 kb downstream of the marker were evaluated. Markers positioned within a gene were further evaluated for predicted transcription factor binding site (TFBS) score differences between major and minor alleles. The online software FABIAN (Steinhaus et al., 2022) was utilized to test query sequences against detailed transcription factor flexible models (TFFM) compiled within the JASPAR 2022 database (Kahn et al., 2018). Query sequences were comprised of 11 bp, including the five nucleotides flanking the marker on the 5′ and 3′ side in the reference genome. Reference sequences were tested with the major allele as the “wild-type” sequence and the minor allele as “variant” sequence. Where applicable, the *Homo sapiens* ortholog of each associated gene was queried through ProteomeHD and STRING databases to identify potential interactions between genes/proteins implicated in the study results (Szklarczyk et al., 2015; Kustatscher et al., 2019).

## 3 Results

### 3.1 Statistical evaluation of wool characteristics

#### 3.1.1 Pearson’s correlation tests for wool characteristics and production traits

Descriptive statistics of wool characteristics are reported for the 313 rams (Table 2), by test location (Supplementary Table S1) and by test year (Supplementary Table S2). Relationships between continuous wool characteristics were evaluated using Pearson correlation tests. The strongest relationship was identified between clean fleece wt. 365 adj. and grease fleece wt. 365 adj. (*r* = 0.83; *p*-value = 3.22e-80) (Table 3). Grease fleece wt. 365 adj. was significantly correlated (*p*-value <.05) with all traits tested. Average fiber diameter was found to have significant positive correlations with both clean and grease fleece 365 adj. weights (*r* = 0.19; *r* = 0.24 and *p*-value = 6.27e-04; *p*-value = 1.55e-05) respectively, and clean fleece wt. 365 adj. had significant positive correlations with staple length 365 adj. (*r* = 0.53; *p*-value = 6.42e-24) and skin wrinkle score (*r* = 0.14; *p*-value = 1.40e-02). Skin wrinkle and face wool scores had a significant positive correlation (*r* = 0.26; *p*-value = 4.09e-06). Clean and grease fleece 365 adj. weights were significantly correlated with initial body weight (*r* = 0.23; *r* = 0.18), final body weight (*r* = 0.35; *r* = 0.43), 140 days ADG (*r* = 0.25; *r* = 0.39) and scrotal circumference (*r* = 0.16; *r* = 0.24).

**TABLE 2:** Descriptive statistics of wool quality characteristics. Wool quality characteristics of the 313 study rams collected from NDSU and UWY central performance ram tests over 3 years.

	Grease fleece wt. 365 adj. (Lb.)	Clean fleece wt. 365 adj. (Lb.)	Staple length 365 adj. (in.)	Average fiber diameter (micron)	Face wool score	Skin wrinkle score
Average ±SD	20.79 ± 3.13	11.82 ± 2.04	5.08 ± 0.57	22.64 ± 1.51	1.30 ± 0.50	1.39 ± 0.45
Min	13.10	7.09	3.10	19.01	1.00	1.00
Median	20.70	11.63	5.09	22.56	1.00	1.25
Max	31.00	18.04	6.97	27.20	3.40	3.50
Range	17.90	10.95	3.87	8.19	2.40	2.50

SD, standard deviation.

**TABLE 3:** Pearson correlation results for ram production and wool characteristics. Correlation coefficients (*r*) are reported on the upper diagonal and *p*-values are reported on the lower diagonal.

	Grease fleece wt. 365 adj.	Clean fleece wt. 365 adj.	Staple length 365 adj.	Face wool score	Skin wrinkle score	Average fiber diameter	SC	Initial weight	Final weight	140 days ADG
Grease Fleece Wt. 365 adj.	—	0.83*	0.41*	0.12*	0.28*	0.24*	0.24*	0.18*	0.43*	0.39*
Clean Fleece Wt. 365 adj.	3.22E-80	—	0.53*	0.09	0.14*	0.19*	0.16*	0.23*	0.35*	0.25*
Staple Length 365 adj.	5.97E-14	6.42E-24	—	0.08	0.16*	−0.04	0.03	0.05	0.28*	0.36*
Face Wool Score	3.41E-02	1.10E-01	1.41E-01	—	0.26*	−0.01	−0.13*	−0.05	−0.01	0.08
Skin Wrinkle Score	4.26E-07	1.40E-02	4.56E-03	4.09E-06	—	0.05	−0.09	−0.04	0.25*	0.45*
Average Fiber Diameter	1.55E-05	6.27E-04	4.42E-01	8.05E-01	3.94E-01	—	0.12*	0.20*	0.16*	0.01
SC	1.65E-05	3.95E-03	6.34E-01	1.93E-02	1.09E-01	3.81E-02	—	0.26*	0.42*	0.26*
Initial Weight	1.10E-03	3.64E-05	3.73E-01	3.59E-01	4.61E-01	3.39E-04	2.37E-06	—	0.66*	−0.05
Final Weight	2.77E-15	2.09E-10	3.50E-07	8.56E-01	8.13E-06	5.97E-03	1.13E-14	6.96E-40	—	0.71*
140 days ADG	4.95E-13	7.89E-06	4.27E-11	1.83E-01	3.91E-17	8.71E-01	3.18E-06	3.68E-01	4.51E-49	—

*indicates a significant *p*-value (<.05). Face wool score and skin wrinkle score were tested as log10 transformed data. 140 days ADG, average daily gain over 140 days; SC, scrotal circumference.

#### 3.1.2 Relationship of wool quality characteristics to presence of belly wool

**FIGURE 1:**
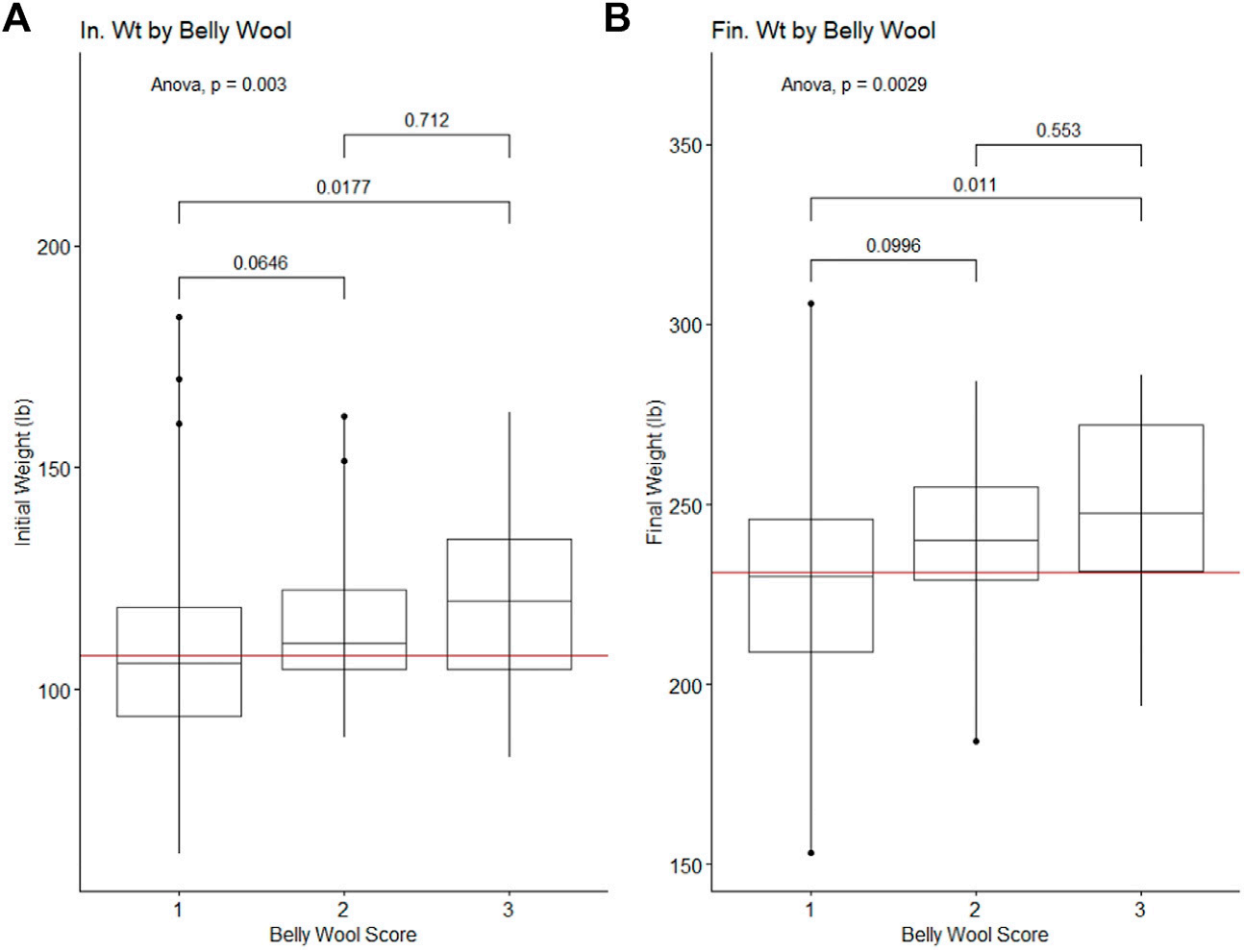
Significant ANOVA results and post-hoc Tukey HSD *p*-values for production traits tested against belly wool categories. **(A)** Initial on-test weight, **(B)** Final test weight. Horizontal red lines indicate the trait mean.

**FIGURE 2:**
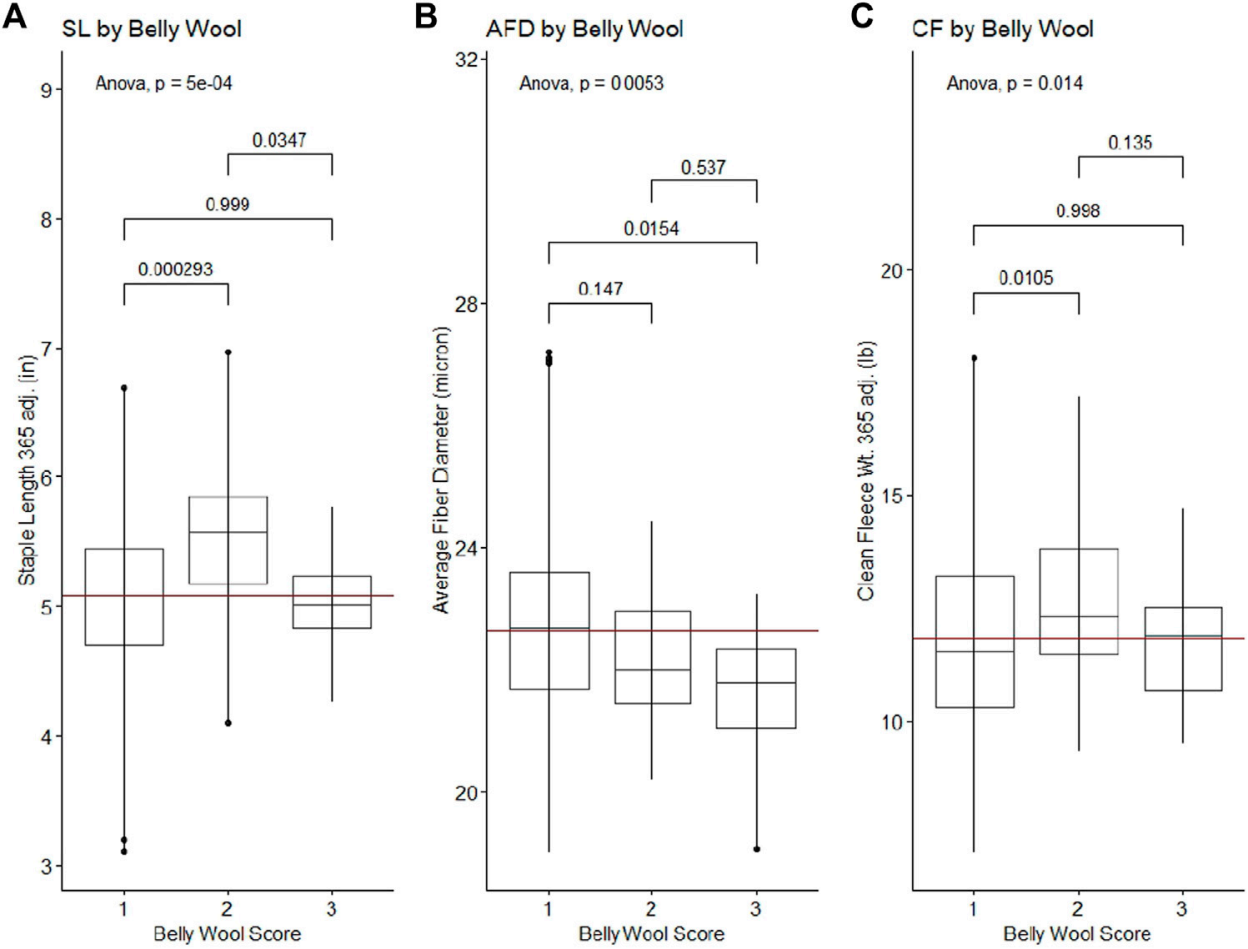
Significant ANOVA results and post-hoc Tukey HSD *p*-values for wool quality characteristics tested against belly wool categories. **(A)** Staple length 365 adj., **(B)** average fiber diameter, **(C)** clean fleece wt. 365 adj. Horizontal red lines indicate the trait mean.

Belly wool score was found to have significant relationships with initial body weight and final body weight, with rams in category three tending to have greater weights than rams in category one (Tukey HSD *p*-value ∼0.01) (Figure 1). Significant relationships were identified with wool characteristics staple length 365 adj. (*p*-value = 5e-04), average fiber diameter (*p*-value = 0.5.3e-3) and clean fleece wt. 365 adj. (*p*-value = 1.4e-2). Post hoc Tukey HSD tests revealed that rams within belly wool category two had significantly longer staple length 365 adj. than category one (*p*-value = 2.93e-4) and category three (*p*-value = 3.47e-2) rams. Rams with belly wool scores in category three had significantly finer average fiber diameter than rams in category one (*p*-value = 1.54e-2), although there was no significant difference between rams in categories three and two or two and one. For clean fleece wt. 365 adj., rams in belly wool category two had significantly greater measurements than rams in category one (*p*-value = 1.05e-2) (Figure 2). The relationships between belly wool and grease fleece wt. 365 adj., face wool score, skin wrinkle score, scrotal circumference and 140 days ADG were also investigated and were not found to be significant.

### 3.2 Principal component analysis

**FIGURE 3:**
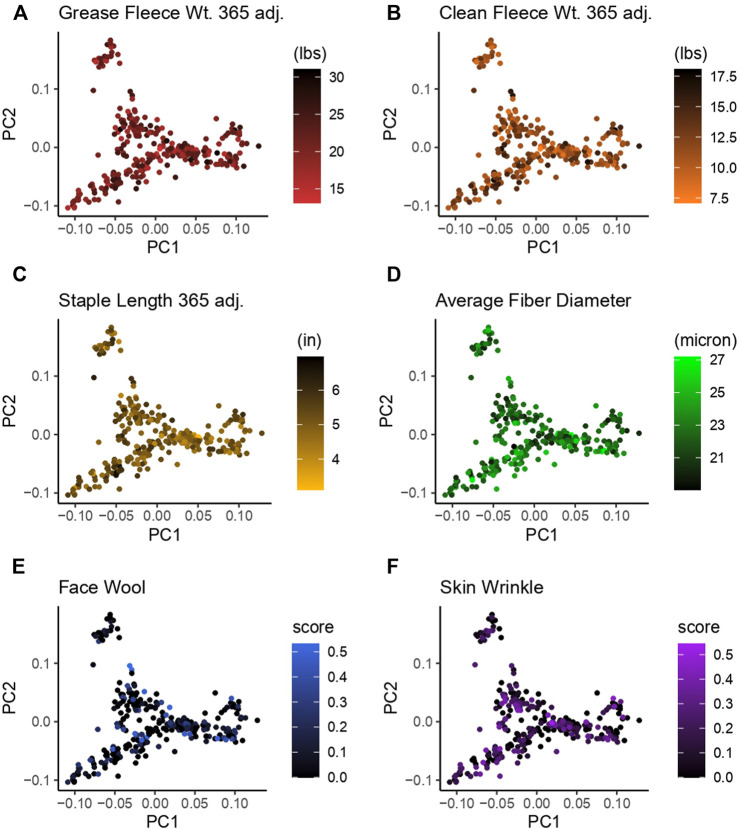
Principal component analysis (PCA) for 313 Rambouillet rams. Each panel represents PC1 plotted on the x-axis and PC2 plotted on the y-axis. Rams are color-coded based on their position within the trait distribution, with the most desirable end of the distribution represented by black. Each panel is color-coded low to high: **(A)** grease fleece wt. 365 adj., red to black; **(B)** clean fleece wt. 365 adj., orange to black; **(C)** staple length 365 adj., yellow to black; **(D)** average fiber diameter, black to green; **(E)** face wool score, black to blue, **(F)** skin wrinkle score, black purple. Face wool and skin wrinkle are colored based on log10 transformed data.

Principal component analysis (PCA) was used to investigate the population structure as it related to wool quality characteristics. Plots were constructed with principal component 1 (PC1) on the x-axis and principal component 2 (PC2) on the y-axis. PC1 had an eigenvalue of 8.57 and explained 11.17% of the total variance, PC2 had an eigenvalue of 6.91 and explained 9.00% of the total variance (Figure 3). There does not appear to be any specific clustering of phenotypically similar rams in the first or second PC for the wool traits examined. Color-coding of rams by wool quality characteristic distributions indicates these specific characteristics do not segregate with any particular genetic relationships.

### 3.3 Genome-wide association studies

**FIGURE 4:**
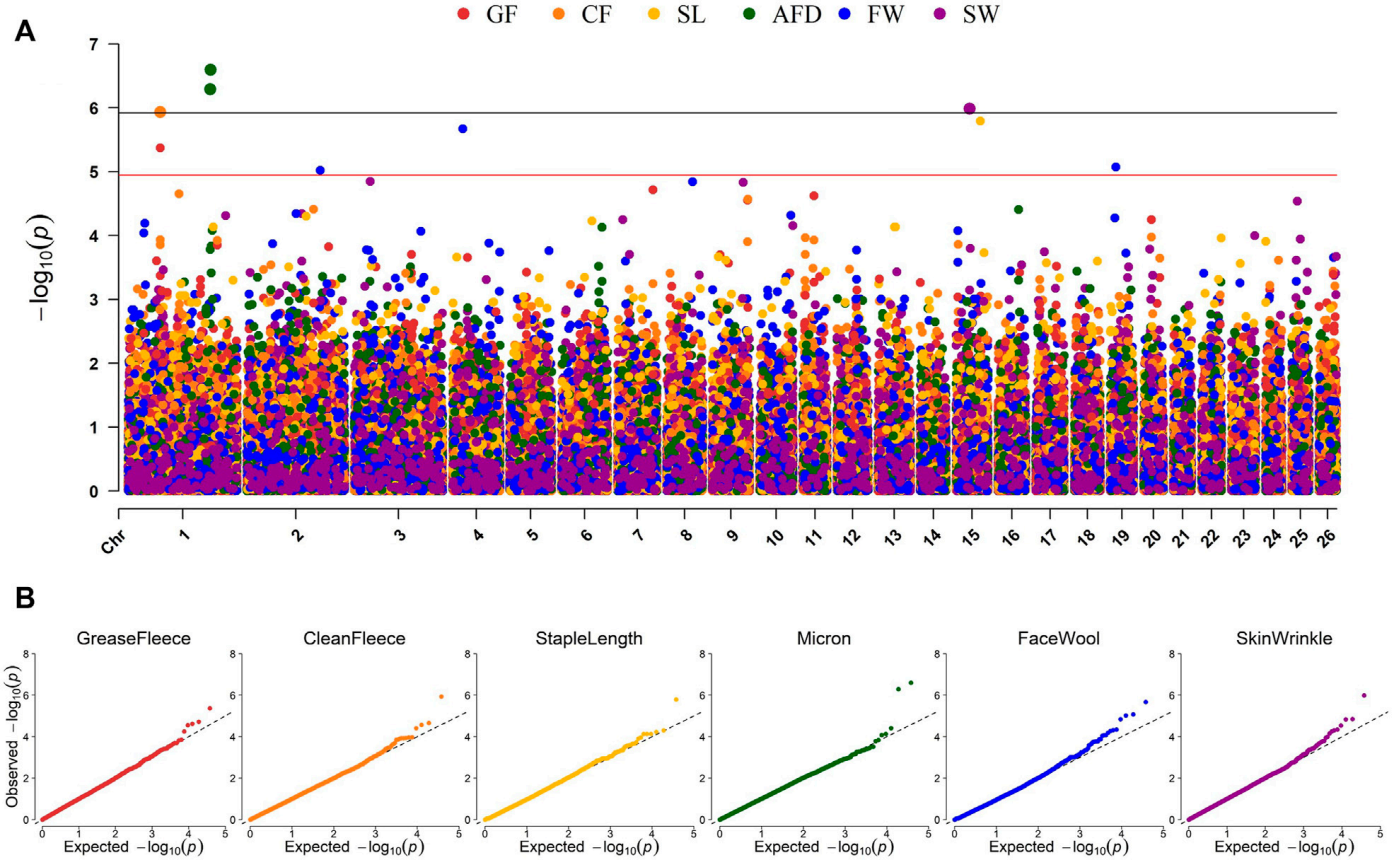
Manhattan and QQ plots representing EMMAX GWAS results for six continuous wool characteristics. **(A)** Manhattan plot representing the GWAS results of six wool traits. *p*-values are represented by: grease fleece wt. 365 adj., red; clean fleece wt. 365 adj., orange; staple length 365 adj., yellow; average fiber diameter, green; face wool score, blue; skin wrinkle score, violet. Genome-wide significance is given by *p*-values <1.19e-06 (black line) and chromosome-wide significance is given by *p*-values <1.13e-05 (red line). **(B)** Quantile-Quantile (QQ) plots for each GWAS displaying the expected *versus* observed–log10 (*p*-value).

Genome-wide association studies (GWAS) were conducted for each of the six continuous wool quality characteristics. Ram test location and/or ram test year were included as fixed effects for traits with significant (*p*-value <0.05) ANOVA or *t*-test results. The results of GWAS are displayed in a multi-trait Manhattan plot (Figure 4A) and individual quantile-quantile (QQ) plots (Figure 4B) and unadjusted *p*-values are reported (Table 4). Three SNPs on chromosome 1 reached genome-wide significance, including two SNPs associated with average fiber diameter and one SNP associated with clean fleece wt. 365 adj. Significant SNPs for average fiber diameter were identified in a dominant inheritance model (rs404487383 with *p*-value = 2.53e-07; rs406184307 with *p*-value = 5.11e-07) and were estimated to explain 8.25% and 7.85% of phenotypic variance. The significant SNP rs420943224 was found to be significant for clean fleece wt. 365 adj. by genome-wide threshold and for grease fleece wt. 365 adj. by chromosome-wide threshold in the corresponding additive inheritance models (rs420943224; *p*-value = 1.16e-06; *p*-value = 4.27e-06) with 7.40% and 6.60% proportion of variance explained (PVE), respectively. Two significant SNPs were identified on chromosome 15 for skin wrinkle score (rs402689377; additive) and staple length (OAR15_66653722.1; recessive) and three significant SNPs on chromosomes 2, 4, and 19 were associated with face wool score in a recessive inheritance model (OAR2_197807108.1; rs429550684; OAR19_14805437.1). The PVE for significant SNPs ranged from 6.13% to 8.25% and MAF ranged from 5.13% to 48.40%.

**TABLE 4:** Results of GWAS for wool quality characteristics. Each trait was tested individually in an EMMAX model and significant markers (genome-wide, *p*-values <1.19e-06; chromosome-wide, *p*-values <1.13e-05) are reported.

Marker ID	rs number	Chr: Position (bp)	Trait	Model	COV	*p*-value	MAF (%)	PVE (%)	Genomic context
OAR1_224418361.1	rs404487383	1:210,457,046	AFD	D	Y	2.53e-07	36.22	8.25	Within 60S ribosomal protein L17-like (LOC121818710) (Yang et al., 2016)
OAR1_224016330.1	rs406184307	1:210,061,545	AFD	D	Y	5.11e-07	48.40	7.85	Downstream of LOC121816904 (lncRNA)
s29455.1	rs402689377	15:34,799,858	SW	A	P, Y	1.03e-06	25.40	7.47	Intronic, ATP binding cassette subfamily C member 8 (ABCC8) (Shorter et al., 2008)
OAR1_86433231.1	rs420943224	1:81,908,905	CF	A	P, Y	1.16e-06	5.13	7.40	Upstream of U6 spliceosomal RNA (LOC114110993) (Hilcenko et al., 2013)
OAR15_66653722.1	—	15:61,931,743	SL	R	P	1.62e-06	16.77	7.16	Downstream of WT1 (Wagner et al., 2008), upstream of LOC105602333, upstream of translation machinery-associated protein 7-like (LOC114118447), upstream of LOC114118448 (lncRNA)
OAR4_26881691.1	rs429550684	4:26,484,846	FW	R	P	2.14e-06	44.63	7.00	Upstream of LOC121819390 (lncRNA), downstream of 40S ribosomal protein S19-like (LOC101106000) (Kuramoto et al., 2005)
OAR1_86433231.1	rs420943224	1:81,908,905	GF	A	P	4.27e-06	5.13	6.60	Upstream of U6 spliceosomal RNA (LOC114110993) (Hilcenko et al., 2013)
OAR19_14805437.1	—	19:14,279,682	FW	R	P	8.49e-06	33.65	6.20	Intronic, ULK4 (unc-51 like Kinase 4) (Luo and Yang 2022)
OAR2_197807108.1	—	2:187,691,398	FW	R	P	9.58e-06	9.97	6.13	Intergenic

Chr, chromosome; BP, base pair position; AFD, average fiber diameter; SW, skin wrinkle score; CF, clean fleece wt. 365 adj.; SL, staple length 365 adj.; FW, face wool score; GF, grease fleece wt. 365 adj.; A, additive inheritance; D, dominant inheritance; R, recessive inheritance; COV, covariate; Y, year; P, place; MAF, minor allele frequency; PVE, proportion of variance explained.

#### 3.3.1 Marker validation through ANCOVA

**FIGURE 5:**
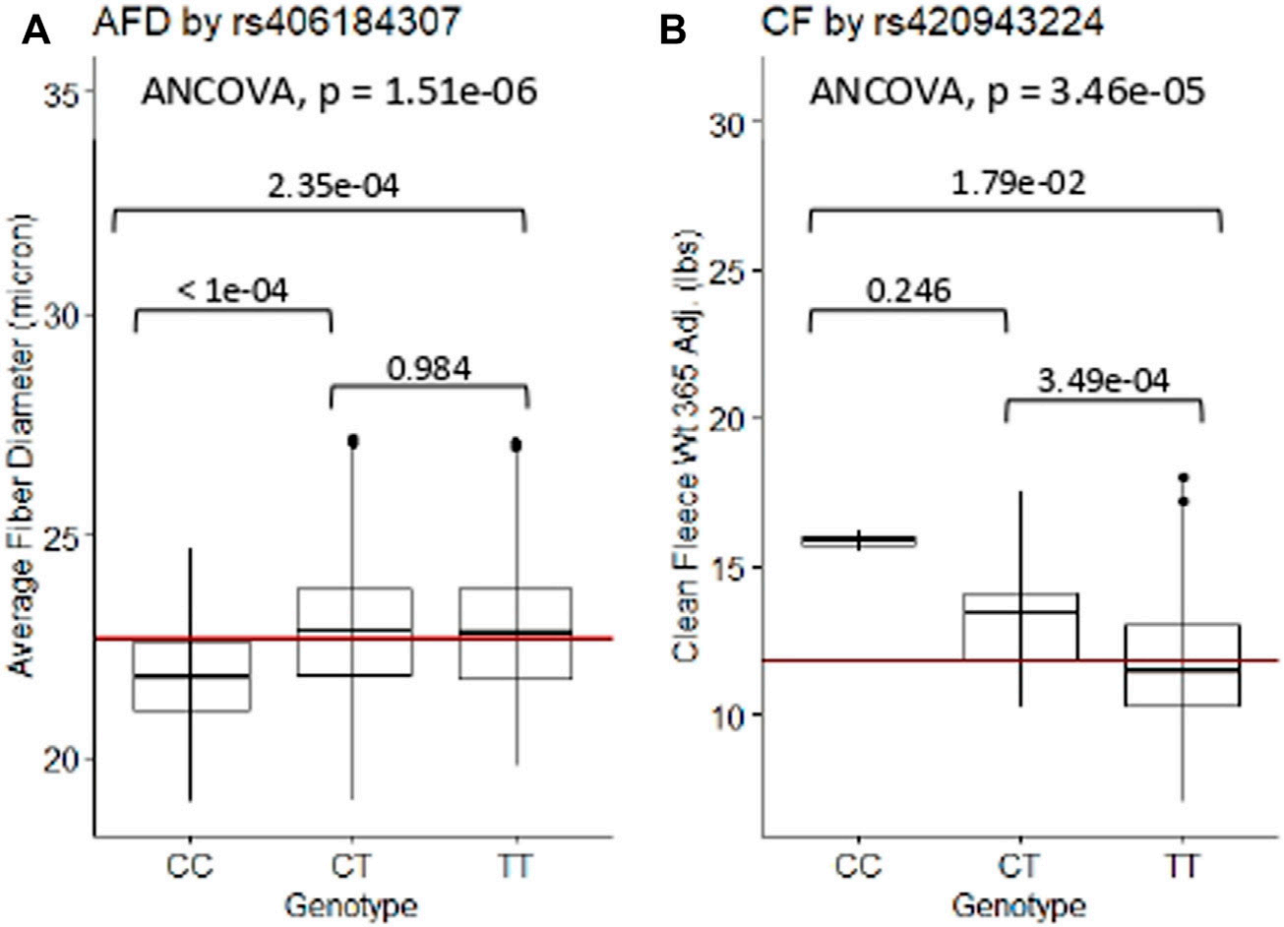
ANCOVA and post-hoc Tukey HSD test results of selected significant SNPs. **(A)** Average fiber diameter against SNP rs406184307, and **(B)** Clean fleece wt. 365 adj. against significant SNP rs420943224. The red horizontal lines indicate the trait means. AFD, average fiber diameter; CF, clean fleece wt. 365 adj.

The relationships between significant GWAS SNP genotypes and their associated wool quality characteristics were further evaluated through ANCOVA and Tukey HSD tests. The mean trait values for the alternate homozygous genotype, heterozygous genotype and reference homozygous genotype of each SNP are reported (Supplementary Table S3). Rams homozygous for the major allele (CC) at rs406184307 were found to have significantly lower average fiber diameter measurements than rams heterozygous (CT; *p*-value < 1e-04) or homozygous for the minor allele (TT; *p*-value = 2.35e-04) (Figure 5A). Presence of one or two copies of the C allele at rs420943224 had significantly greater mean clean fleece wt. 365 adj. than rams homozygous for the major allele (TT) with *p*-value = 3.49e-04 and 1.79e-02, respectively (Figure 5B). Boxplot figures for remaining significant SNPs are located in Supplementary Figures S1, S2.

### 3.4 Genomic context of significant markers

To investigate the genomic context of GWAS results, the reference genome sequence was evaluated for the presence of known or predicted genes (Table 4). The functional consequences of SNPs within genes were further investigated through TFBS prediction analysis. Reference genome sequence for the markers rs404487383 within 60S ribosomal protein L17-like (LOC121818710), rs402689377 within ATP binding cassette subfamily C member 8 (*ABCC8*) and OAR19_14805437.1 within unc-51 like Kinase 4 (*ULK4*) were queried for TFBS differences. Score difference between reference and alternate allele sequences of 0.3 or −0.3 or greater are recorded (Table 5). Four SOX family TFBS and a TCF7 TFBS were predicted at rs404487383. Three TFBS with a score difference of +3/-3 or greater were predicted at both rs402689377 and SNP OAR19_14805437.1. Query of *Homo sapiens* ortholog genes through ProteomeHD and STRING databases revealed a known interaction between human proteins RPL17 and RPS19 with a co-regulation percentile score of 0.9998 (https://www.proteomehd.net/proteomehd/P18621/0.989988).

**TABLE 5:** Predicted TFBS for SNPs located within genes. Query sequences were analyzed with the major allele as “wild type” and the minor allele as “variant” sequence. The score depicts the difference of wild type versus variant predictiosns.

Marker ID	Predicted TFBS	Score	Query
rs404487383	SOX10	−0.90	TCTTT[T/C]GTTGC
	SOX2	−0.50	
	SOX2	−0.49	
	SOX17	−0.45	
	TCF7	−0.32	
rs402689377	ETS2	−0.56	CTTTC[C/T]GGCTC
	TFCP2	−0.32	
	RUNX2	0.55	
OAR19_14805437.1	MEIS1	0.33	AGTGA[T/C]TCTGG
	MEIS2	0.41	
	NR2F2	0.58	

## 4 Discussion

To the authors’ knowledge, this study represents the first GWAS conducted for wool quality characteristics of Rambouillet sheep. Genetic markers for wool quality traits have been previously identified through GWAS for other breeds of sheep, including Merino and Chinese fine-wool sheep (Wang et al., 2014; Zhao et al., 2021a; Bolormaa et al., 2021; Zhao B. et al., 2021), North-Caucasian sheep (Krivoruchko et al., 2022) and Baluchi sheep (Ebrahimi et al., 2017). Of note, the marker rs410503867 reported for association with super-elite rams (Krivoruchko et al., 2022) was positioned 2.3 Mb from a marker significant for face wool (rs429550684) in the current study. Additionally, Zhao et al. (2021a) reported markers within candidate genes *USP13* and *NLGN1* associated with staple length and positioned 1.8 Mb and 3.3 Mb, respectively, from markers identified for average fiber diameter (rs404487383; rs406184307) in the present study. Genetic markers for wool quality in sheep have also been suggested through candidate gene studies, including markers associated with genes *MTR* (Rong et al., 2015), *FST* (Ma et al., 2017), *DKK1* (Mu et al., 2017), *KIF16B* (Zhao et al., 2021c), *FGF5* (Zhao et al., 2021d) and keratin-associated proteins (Gong et al., 2016; Itenge, 2021). In candidate gene studies within the Rambouillet breed, several keratin intermediate filament (*KRT*) and keratin-associated protein (*KAP*) genes have been suggested for genomic selection (Mahajan et al., 2017a; Mahajan et al., 2017b; Mahajan et al., 2019; Singh et al., 2022). Despite this body of literature, there is still a need for robust genome-wide investigations for markers associated with wool characteristics of Rambouillet sheep.

**FIGURE 6:**
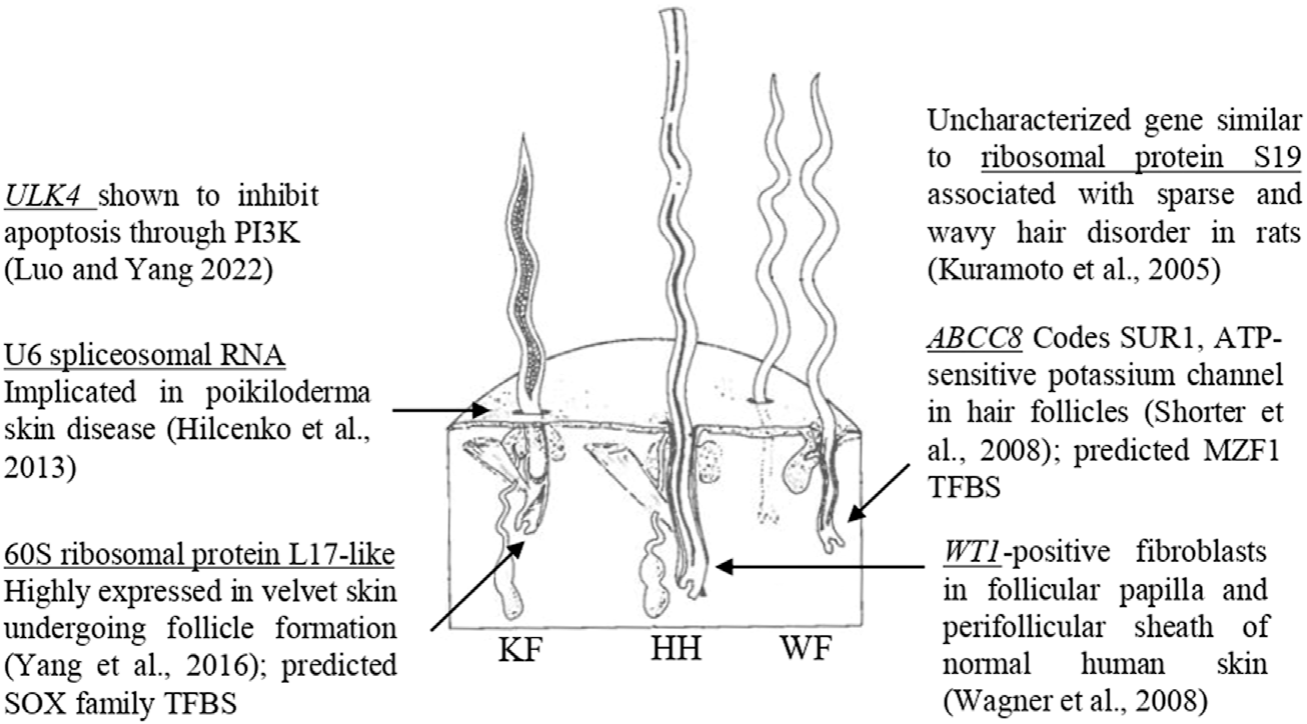
Genes with biological functions relevant to follicular growth. Significant SNPs identified in GWAS are located within or near genes with known biological roles relevant to skin and follicular growth, including ULK4 (**Luo and Yang, 2022**), U6 spliceosomal RNA (**Hilcenko et al., 2013**), 60S ribosomal protein L17-like (**Yang et al., 2016**), ribosomal protein S19 (**Kuramoto et al., 2005**), ABCC8 (**Shorter et al., 2008**) and WT1 (**Wagner et al., 2008**). The figure illustrates the three types of fibers which comprise sheep’s wool, adapted from **Bradford and Fitzhugh (1983)**. KF, kemp fiber; HH, heterotype hair; WF, wool fiber.

The current study investigated a genome-wide distribution of SNP markers for significance against six wool quality characteristics. Of the eight significant SNPs identified, seven markers were located in proximity to at least one gene, and three of these markers were located within a gene. Genes containing significant SNPs have been previously associated with biological functions relevant to follicle growth (Figure 6). The marker rs402689377 associated with skin wrinkle score was located within the gene ATP binding cassette subfamily C member 8 (*ABCC8*) which codes for SUR1, an ATP-sensitive potassium channel known to affect fiber growth (Shorter et al., 2008). Although *ABCC8* has not been previously associated with wool quality in sheep, another potassium voltage-gated channel-related gene (*KCNIP4*) has been suggested to be related to sheep growth (Mohammadi et al., 2020). The marker OAR19_14805437.1 associated with face wool score was within unc-51 like Kinase 4 (*ULK4*). Overexpression of the gene *ULK4* has been shown to inhibit apoptosis (Luo and Yang 2022). Fiber producing follicles undergo a cycle including active growth, apoptosis-driven involution (catagen), shedding and resting in humans and mice (Botchkareva et al., 2006). The wool follicles of sheep are known to undergo similar cyclic activity with periods of catagen (Liu et al., 2013). The most significant SNP for average fiber diameter, rs404487383, was within 60S ribosomal protein L17-like. This gene was previously found to be one of the 50 most highly expressed genes within regenerating velvet skin of Red deer (Yang et al., 2016), suggesting a potential role in the sheep skin transcriptome.

Significant SNPs were located in regions containing biologically relevant genes. The marker rs429550684 was significant for face wool score and was located downstream of 40S ribosomal protein S19-like (LOC121818710). The gene similar to ribosomal protein S19 (LOC364797) was identified within the sparse and wavy hair (*swh*) locus of rats (Kuramoto et al., 2005), although annotation for this gene has since been withdrawn from the *Rattus norvegicus* assembly as it was not predicted in a later annotation (NCBI Gene ID: 364797). The *Homo sapiens* orthologs of 40S ribosomal protein S19-like and 60S ribosomal protein L17-like have known protein interactions, suggesting a potential for similar interaction of these proteins in sheep. The *swh* locus is known to be associated with follicle hypoplasia, as well as impaired development of the sebaceous glands and mammary glands (Kuramoto et al., 2005). The marker rs420943224 associated with clean fleece wt. 365 adj. and grease fleece wt. 365 adj. was located upstream of U6 spliceosomal RNA (LOC114110993). A spliceosomal U6 small nuclear RNA has been previously indicated in poikiloderma with neutropenia (Hilcenko et al., 2013). Finally, the marker OAR15_66653722.1 associated with staple length 365 adj. was located downstream of *WT1*, which has been identified in fibroblast cells that can induce and support hair growth (Wagner et al., 2008). The proximity of significant SNPs to these genes suggests the possibility for linkage disequilibrium with untested causative markers, or the possibility for identified SNPs to be positioned within transcriptional regulatory elements. Further work is needed to elucidate the implications of these associations.

Prediction analysis for TFBS suggested binding motifs for SOX2, SOX3, SOX10 and/or SOX17 may have less specific binding abilities between the alternate and reference alleles at rs404487383. SOX2 is expressed in mesenchymal cells during skin development (Sarkar and Hochedlinger, 2013), and both SOX2 and SOX3 are involved in the development of inner-ear hair cells in zebrafish (Gou et al., 2018). The gene SOX10 has been shown to play an important role in the development of vestibular hair cells in the pig (Qi et al., 2022). These data suggest potential functional ramifications of variant alleles associated with wool quality characteristics.

The trait correlations reported in the current study largely agree with previously published work for Rambouillet sheep. Clean fleece wt. 365 adj. is a component of grease fleece wt. 365 adj., which is reflected in their robust correlation in this study and previous (Vesely et al., 1970). Clean fleece wt. 365 adj. was significantly correlated with both staple length 365 adj. and average fiber diameter, which agrees with relationships previously reported for clean fleece and fiber diameter (Lupton et al., 2002; Hanford et al., 2005; Mahajan et al., 2018). This study found no significant correlation between staple length 365 adj. and average fiber diameter, although staple length and fiber diameter (wool grade) have been previously reported to be either favorably or unfavorably related (Lupton et al., 2002; Hanford et al., 2005). There was a significant positive relationship between clean fleece wt. 365 adj. and scrotal circumference, which was similar to observations made in Merino rams and in other central performance ram tests (Duguma et al., 2002; Lupton et al., 2002). The presence of significant correlations between wool quality characteristics indicates that progress in one trait may either positively or negatively impact progress in another trait; for instance, gains in clean fleece weight may come at the expense of fiber diameter.

Similar associations as those reported between belly wool category and wool quality characteristics in the present study have been previously noted in Merino sheep (Naidoo et al., 2016). Genetic correlation between wool quality and ‘creeping belly’ scores, representing the extent of belly-type wool on the side of the sheep, were reported to be −0.55 ± 0.27. This relationship was interpreted as sheep with more acceptable wool quality also tended to have less acceptable scores for creeping belly. Creeping belly has been reported to be correlated with body weight in Afrino sheep (Snyman and Olivier, 2002), and importantly, has been reported to have an unfavorable correlation with reproduction (Snyman and Olivier, 2002). The current study suggested potential positive phenotypic associations between belly score category two and staple length 365 adj., average fiber diameter, clean fleece wt. 365 adj. and ram initial and final body weights, although further evaluation is needed to understand other important associations with belly wool in Rambouillet sheep.

The PCA investigated in this study indicated an overall lack of segregation of genetically similar animals with any of the specific wool quality characteristics tested by PC1 or PC2, suggesting an opportunity for genetic progress for wool quality characteristics. Previously reported heritabilities suggest that genetics are a major factor in wool quality phenotypes and genetic improvements should be possible even in the short term (Medrado et al., 2021). This study suggests specific genetic markers that may be used in marker-assisted selection programs for wool quality in Rambouillet sheep to make gains in economically important traits such as average fiber diameter and clean fleece weight. Interpretation of the current study is somewhat limited by sample size, as some genotype categories (such as CC at rs420943224) have as few as two rams observed. Improving sample sizes in underrepresented genotypes would improve statistical power and overall understanding of genotypic relationships with traits.

## Data Availability

The datasets have been deposited to EVA repository: https://urldefense.com/v3/__https://www.ebi.ac.uk/eva/?eva-study=PRJEB58836__;!!JYXjzlvb!gEHSLvJ5dvR-1g4UW6svxSQlbb8v0CZDShYTgf1HgGPLj3rxwSZ0_u0CaQFKVS5cKDCOr7UFqZw7zvCuQZ4qjmD-Q$ Accession Details are: Project: PRJEB58836, Analyses: ERZ15609617.

## Abbreviations

365 adj., adjusted to 365 days; CO, Colorado; MT, Montana; ND, North Dakota; SD, South Dakota; WY, Wyoming.
