# Corrigendum: Genetic association of wool quality characteristics in United States Rambouillet sheep

**DOI:** 10.3389/fgene.2024.1471185

**Published:** 2024-09-05

**Authors:** Gabrielle M. Becker, Julia L. Woods, Christopher S. Schauer, Whit C. Stewart, Brenda M. Murdoch

**Affiliations:** ^1^ Department of Animal, Veterinary and Food Sciences, University of Idaho, Moscow, ID, United States; ^2^ Hettinger Research Extension Center, North Dakota State University, Hettinger, ND, United States; ^3^ Department of Animal Science, University of Wyoming, Laramie, WY, United States

**Keywords:** 60S ribosomal protein L17-like, ABCC8, central performance ram test, GWAS, sheep production

In the published article, there were errors in **Results**, **Discussion**, Table 5 and Figure 6. The details of these errors are given below:

**TABLE 5:** Predicted TFBS for SNPs located within genes. Query sequences were analyzed with the major allele as “wild type” and the minor allele as “variant” sequence. The score depicts the difference of wild type versus variant predictiosns.

Marker ID	Predicted TFBS	Score	Query
rs404487383	SOX10	−0.90	TCTTT[T/C]GTTGC
	SOX2	−0.50	
	SOX2	−0.49	
	SOX17	−0.45	
	TCF7	−0.32	
rs402689377	ETS2	−0.56	CTTTC[C/T]GGCTC
	TFCP2	−0.32	
	RUNX2	0.55	
OAR19_14805437.1	MEIS1	0.33	AGTGA[T/C]TCTGG
	MEIS2	0.41	
	NR2F2	0.58	

**FIGURE 6:**
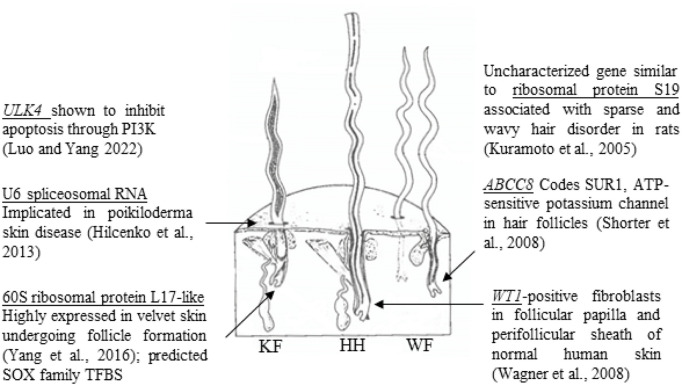
Genes with biological functions relevant to follicular growth. Significant SNPs identified in GWAS are located within or near genes with known biological roles relevant to skin and follicular growth, including ULK4 (**Luo and Yang, 2022**), U6 spliceosomal RNA (**Hilcenko et al., 2013**), 60S ribosomal protein L17-like (**Yang et al., 2016**), ribosomal protein S19 (**Kuramoto et al., 2005**), ABCC8 (**Shorter et al., 2008**) and WT1 (**Wagner et al., 2008**). The figure illustrates the three types of fibers which comprise sheep’s wool, adapted from **Bradford and Fitzhugh (1983)**. KF, kemp fiber; HH, heterotype hair; WF, wool fiber.

A correction has been made to **3 Results**, *3.4 Genomic context of significant markers*, paragraph 1. This sentence previously stated:

“Four SOX family TFBS were predicted at rs404487383 and a YY2 and MZF1 TFBS were predicted at rs402689377. There were no TFBS with score difference of 3/-3 or greater predicted at SNP OAR19_14805437.1.”

The corrected sentence appears below:

“Four SOX family TFBS and a TCF7 TFBS were predicted at rs404487383. Three TFBS with a score difference of +3/-3 or greater were predicted at both rs402689377 and SNP OAR19_14805437.1.”

A correction has been made to **4 Discussion**, paragraph 4. This sentence previously stated:

“Two potential TFBS were predicted with matrix score changes between SNP alternate and reference alleles at s29455.1, a marker associated with skin wrinkle scores. The TF MZF1 has been shown to diminish the expression of the gene PADI1 in human keratinocyte cells (**Dong et al., 2008**). These *in silico* analyses suggest functional ramifications of variant alleles associated with wool quality characteristics.”

The corrected sentence appears below:

“These data suggest potential functional ramifications of variant alleles associated with wool quality characteristics.”

There was an error in [Table 5] as published. [The alternate allele used in the query sequence of rs404487383 and OAR19_14805437.1 were incorrect, and the query sequence of rs402689377 was given for the non-coding strand. The corrections of these errors result in the following changes: for rs404487383, the SOX10 score is corrected from −0.91 to −0.90, the SOX17 score is corrected from −0.39 to −0.45, and the transcription factor TCF7 is included with a score of −0.32; for rs402689377, the transcription factors YY2 and MZF1 are removed and ETS2 (score −0.56), TFCP2 (score −0.32), and RUNX2 (score 0.55) are included; and for OAR19_14805437.1, the transcription factors MEIS1 (score 0.33), MEIS2 (score 0.41), and NR2F2 (score 0.58) are included.]. The corrected [Table 5] and its caption **[Predicted TFBS for SNPs located within genes. Query sequences were analyzed with the major allele as “wild type” and the minor allele as “variant” sequence. The score depicts the difference of wild type versus variant predictions.] appear below.

There was an error in [Figure 6] as published. [The transcription factor MZF1 was incorrectly referenced in this figure]. The corrected [Figure 6] and its caption **[Genes with biological functions relevant to follicular growth. Significant SNPs identified in GWAS are located within or near genes with known biological roles relevant to skin and follicular growth, including ULK4 (Luo and Yang, 2022), U6 spliceosomal RNA (**Hilcenko et al., 2013**), 60S ribosomal protein L17-like (**Yang et al., 2016**), ribosomal protein S19 (**Kuramoto et al., 2005**), ABCC8 (**Shorter et al., 2008**) and WT1 (**Wagner et al., 2008**). The significant SNPs within 60S ribosomal protein L17-like and ABCC8 had predicted TFBS score differences between reference and alternate alleles. The figure illustrates the three types of fibers which comprise sheep’s wool, adapted from **Bradford and Fitzhugh (1983)**. KF, kemp fiber; HH, heterotype hair; WF, wool fiber.] appear below.

The authors apologize for these errors and state that this does not change the scientific conclusions of the article in any way. The original article has been updated.

