# Supplementary material for: Genetic association of wool quality characteristics in United States Rambouillet sheep
Source: Front Genet. 2023 Jan 23;13:1081175. doi: 10.3389/fgene.2022.1081175 (PMC9901206; doi:10.3389/fgene.2022.1081175)
Supplement: Supplementary file 1 [file Table1.DOCX]

Supplementary Material

# Supplementary Data

## Supplementary Figures

**Supplementary Table 1.** Descriptive statistics of wool quality characteristics by central performance ram test location. Face wool score and skin wrinkle score data were transformed with a log10 transformation to improve normality and grease fleece weight, clean fleece weight and staple length measurements were adjusted to 365-days. NDSU, North Dakota State University; UWY, University of Wyoming.

|  |  | Grease Fleece Wt. 365 adj. (lb) | Clean Fleece Wt. 365 adj. (lb) | Staple Length 365 adj. (in) | Average Fiber Diameter (micron) | Face Wool Score | Skin Wrinkle Score |
| --- | --- | --- | --- | --- | --- | --- | --- |
| NDSU | Average ± SD | 20.17 ± 3.14 | 11.45 ± 2.06 | 4.87 ± 0.50 | 22.72 ± 1.51 | 1.17 ± 0.31 | 1.10 ± 0.24 |
|  | Min | 14.24 | 7.23 | 3.10 | 19.01 | 1.00 | 1.00 |
|  | Median | 20.20 | 11.29 | 5.00 | 22.65 | 1.00 | 1.00 |
|  | Max | 30.42 | 18.04 | 5.50 | 27.11 | 3.00 | 2.50 |
|  | Range | 16.18 | 10.81 | 2.40 | 8.10 | 2.00 | 1.50 |
| UWY | Average ± SD | 21.36 ± 3.02 | 12.15 ± 1.97 | 5.27 ± 0.56 | 22.56 ± 1.50 | 1.41 ± 0.60 | 1.65 ± 0.43 |
|  | Min | 13.10 | 7.09 | 3.83 | 19.06 | 1.00 | 1.00 |
|  | Median | 21.20 | 11.89 | 5.24 | 22.48 | 1.10 | 1.60 |
|  | Max | 31.00 | 17.54 | 6.97 | 27.20 | 3.40 | 3.50 |
|  | Range | 17.90 | 10.45 | 3.14 | 8.14 | 2.40 | 2.50 |

**Supplementary Table 2.** Descriptive statistics of wool quality characteristics by central performance ram test year. Face wool score and skin wrinkle score data were transformed with a log10 transformation to improve normality and grease fleece weight, clean fleece weight and staple length measurements were adjusted to 365-days.

|  |  | Grease Fleece Wt. 365 adj. (lb) | Clean Fleece Wt. 365 adj. (lb) | Staple Length 365 adj. (in) | Average Fiber Diameter (micron) | Face Wool Score | Skin Wrinkle Score |
| --- | --- | --- | --- | --- | --- | --- | --- |
| 2019-2021 | Average ± SD | 20.66 ± 3.18 | 12.17 ± 2.24 | 5.04 ± 0.50 | 22.29 ± 1.32 | 1.28 ± 0.49 | 1.41 ± 0.38 |
|  | Min | 13.10 | 7.09 | 3.83 | 19.06 | 1.00 | 1.00 |
|  | Median | 20.50 | 11.89 | 5.01 | 22.27 | 1.00 | 1.40 |
|  | Max | 29.90 | 18.04 | 6.14 | 25.93 | 3.40 | 2.40 |
|  | Range | 16.80 | 10.95 | 2.31 | 6.87 | 2.40 | 1.40 |
| 2020-2021 | Average ± SD | 20.73 ± 2.67 | 12.05 ± 1.75 | 5.18 ± 0.78 | 22.73 ± 1.49 | 1.39 ± 0.60 | 1.29 ± 0.44 |
|  | Min | 15.20 | 7.86 | 3.10 | 19.01 | 1.00 | 1.00 |
|  | Median | 20.70 | 11.90 | 5.43 | 22.80 | 1.05 | 1.10 |
|  | Max | 26.80 | 16.17 | 6.97 | 27.11 | 3.20 | 3.50 |
|  | Range | 11.60 | 8.31 | 3.87 | 8.10 | 2.20 | 2.50 |
| 2021-2022 | Average ± SD | 20.98 ± 3.43 | 11.27 ± 1.93 | 5.03 ± 0.41 | 22.93 ± 1.64 | 1.24 ± 0.41 | 1.44 ± 0.51 |
|  | Min | 14.24 | 7.23 | 3.88 | 20.04 | 1.00 | 1.00 |
|  | Median | 20.66 | 10.99 | 5.09 | 22.80 | 1.00 | 1.20 |
|  | Max | 31.00 | 15.39 | 6.32 | 27.20 | 2.90 | 2.70 |
|  | Range | 16.76 | 8.16 | 2.44 | 7.16 | 1.90 | 1.70 |

**Supplementary Table 3.** Trait means given by GWAS SNP genotype for each trait. Micron, mean fiber diameter given in microns; skin wrinkle, log10 of score; clean fleece, pounds; staple length, inches; face wool, log10 of score; grease fleece, pounds.

| Marker ID | Trait | Minor Allele | Alt Genotype Mean | Het Genotype Mean | Ref Genotype Mean |
| --- | --- | --- | --- | --- | --- |
| OAR1_224418361.1 | Micron | A | 22.52 | 23.06 | 22.13 |
| OAR1_224016330.1 | Micron | T | 22.92 | 22.88 | 21.87 |
| s29455.1 | Skin Wrinkle | C | 0.21 | 0.14 | 0.10 |
| OAR1_86433231.1 | Clean Fleece | C | 15.85 | 13.32 | 11.64 |
| OAR15_66653722.1 | Staple Length | T | 4.16 | 5.12 | 5.09 |
| OAR4_26881691.1 | Face Wool | T | 0.16 | 0.08 | 0.06 |
| OAR1_86433231.1 | Grease Fleece | C | 23.85 | 23.46 | 20.51 |
| OAR19_14805437.1 | Face Wool | A | 0.17 | 0.09 | 0.07 |
| OAR2_197807108.1 | Face Wool | C | 0.53 | 0.09 | 0.09 |

**
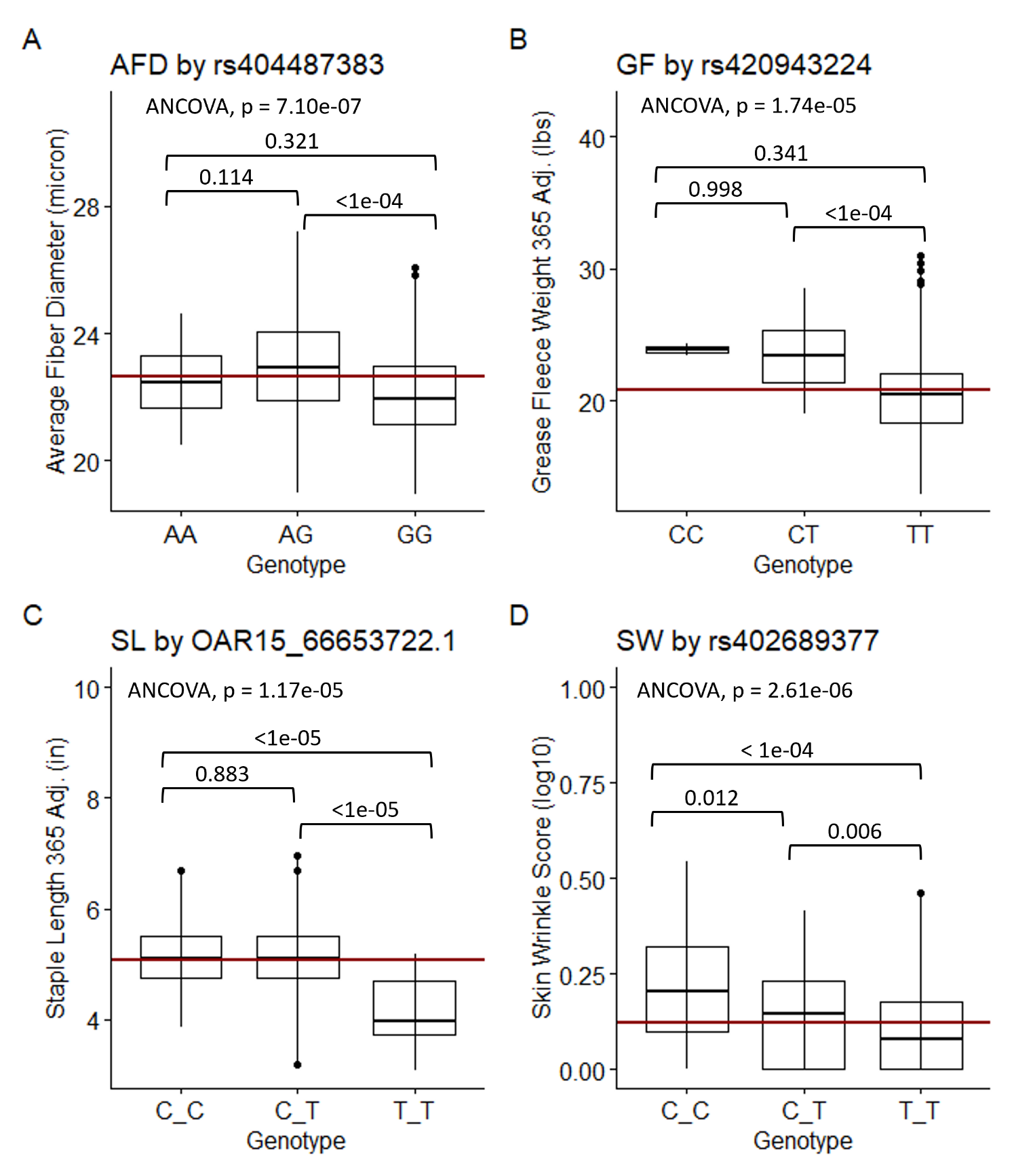
Supplementary Figure 1.** ANCOVA and post-hoc Tukey HSD test results of significant SNPs for average fiber diameter, grease fleece weight, staple length and skin wrinkle score. (A) Average fiber diameter against SNP rs404487383, (B) Grease fleece weight 365 adj. against significant SNP rs420943224, (C) Staple length 365 adj. against OAR15_66653722.1, and (D) Skin wrinkle (log10) by rs402689377. The red horizontal lines of each plot indicate the trait means. AFD, average fiber diameter; GF, grease fleece; SL, staple length; SW, skin wrinkle.


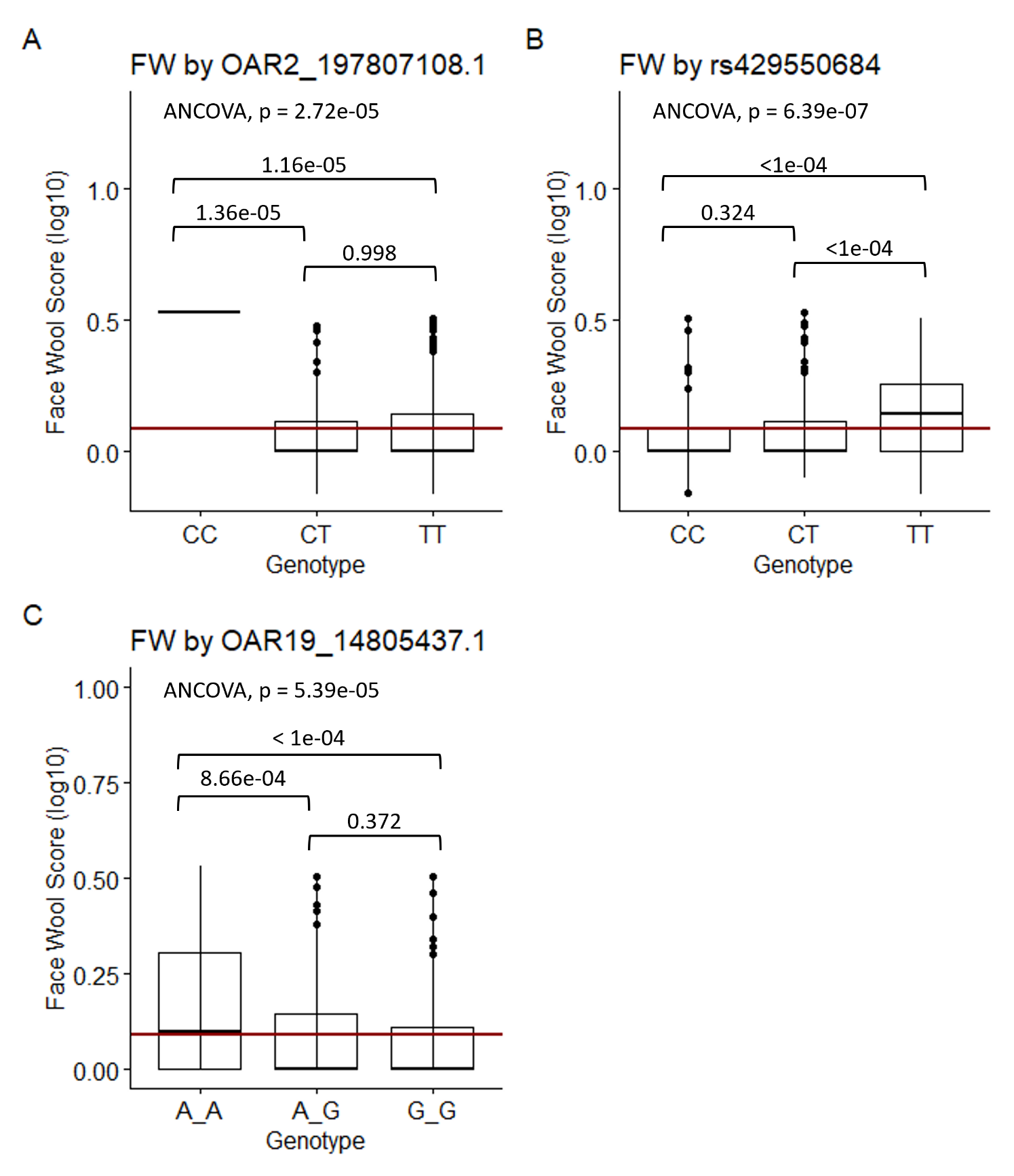
**Supplementary Figure 2** ANCOVA and post-hoc Tukey HSD test results of significant SNPs for face wool. Face wool score against (A) OAR2_197807108.1, (B) rs429550684 and (C) OAR19_14805437.1. The red horizontal lines indicate the trait means. FW, face wool.
